# Interleukin-17A Exacerbates Ferric Chloride-Induced Arterial Thrombosis in Rat Carotid Artery

**DOI:** 10.1155/2014/247503

**Published:** 2014-04-03

**Authors:** Francesco Maione, Antonio Parisi, Elisabetta Caiazzo, Silvana Morello, Fulvio D'Acquisto, Nicola Mascolo, Carla Cicala

**Affiliations:** ^1^Department of Pharmacy, University of Naples Federico II, Via Domenico Montesano 49, 80131 Naples, Italy; ^2^Department of Pharmaceutical and Biomedical Sciences, University of Salerno, Via Ponte Don Melillo, Fisciano, 84084 Salerno, Italy; ^3^William Harvey Research Institute, Barts and The London School of Medicine and Dentistry, Queen Mary University of London, Charterhouse Square, London EC1M 6BQ, UK

## Abstract

Interleukin-17A (IL-17A), the most widely studied member of the IL-17 cytokine family, is a cytokine which emerged to be critical for host defense as well as in the pathogenesis of autoimmune disorders. Moreover, IL-17A is involved in the pathogenesis of cardiovascular diseases, such as atherosclerosis and acute coronary syndrome and in the cardiovascular risk associated with systemic immunological disorders. Consistent with this, we have recently shown that IL-17A increases human and murine platelet response to ADP. In this study we expanded our previous observation and we describe for the first time an *in vivo* prothrombotic effect of the cytokine. Our results show that IL-17A is synergic with a low FeCl_3_ concentration in inducing carotid thrombus in rats and suggest that the effect is likely related to a downregulation of CD39 vascular expression and hydrolyzing activity. Our findings indicate that IL-17A might be an important molecule at the interface between hemostasis and inflammation.

*“This paper is dedicated to the memory of Professor Alfredo Colonna”*

## 1. Introduction

Increasing experimental and clinical evidence show that thrombosis and atherosclerosis might be closely associated with an inflammatory reaction [1]. Inflammation decreases the activity of natural anticoagulant mechanisms, initiates clotting, and impairs the fibrinolytic system. Indeed, proinflammatory molecules are involved in the activation and migration of leukocytes to sites of vascular injury and inflammation and may contribute to the release by activated cells of prothrombotic factors which, in turn, may activate platelets and other cell types [2, 3].

Recently, much attention has been driven on haemostatic disorders observed in subjects affected by immunological chronic inflammation, such as rheumatoid arthritis (RA), multiple sclerosis (MS), and inflammatory bowel disease (IBD) that may represent the cause of the increased cardiovascular risk observed in these pathologies [4–6]. Multiple factors may be implicated. Circulating cytokines and recruited inflammatory cells could cause endothelial activation and dysfunction leading toward a prothrombotic state [7, 8].

Interleukin-17A (IL-17A) is the most widely studied member of the IL-17 cytokine family. It is mainly produced by T-helper (Th)-17 lymphocytes, and also by natural killer T (NKT) cells, *γδ* T cells (*γδ*-17), cytotoxic CD8^+^ T cells (Tc17), and neutrophils [9–11]. IL-17A is a proinflammatory cytokine [12], highly produced in patients with chronic inflammatory diseases, such as RA, MS, and IBD [13–15]. IL-17A is also involved in atherosclerosis [16]; furthermore, in humans a positive correlation has also been found between circulating IL-17A levels and acute coronary syndrome [17, 18]. These findings have suggested that IL-17A might play a role in the cardiovascular risk associated with systemic immunological disorders. Recently, in the attempt of finding a link between inflammatory markers and endothelial dysfunction, Marder et al. [19] demonstrated that IL-17A is an inflammatory marker that could be positively correlated with markers of impaired vascular function in subjects affected by rheumatoid arthritis. Consistent with this, it has also been shown that, on HUVEC, IL-17A reduces CD39 mRNA expression and synergistically with tumour necrosis factor *α* (TNF*α*) it is able to induce tissue factor (TF) expression and to downregulate thrombomodulin expression [20]. It is known that arterial thrombosis is primarily caused by platelet adhesion to the damaged vessel and activation [21]. Following activation, endothelium loses its antithrombotic properties and platelets may become activated [2]. Thus, IL-17A could activate endothelial cells toward a prothrombotic state; however, up to now there is no* in vivo* evidence for this. We have recently demonstrated that IL-17A increases human and murine platelet response to adenosine-5′-diphosphate (ADP).* In vitro* this effect is associated with an increased exposition of P-selectin on platelet surface [22]. Since platelets are primary involved in arterial thrombus formation [21] and an increased platelet reactivity has been found associated with several systemic immunological disorders where also IL-17A is involved [6, 7, 23], here we sought to investigate the effect of IL-17A on* in vivo* arterial thrombus formation.

## 2. Materials and Methods

### 2.1. Ferric Chloride-Induced Thrombosis

Male Wistar rats (300–350 g; Harlan Nossan, Correzzana, MI, Italy) were used for all experiments. Animals were kept under standard conditions with food* ad libitum* and maintained in a 12 h/12 h light/dark cycle at 22 ± 1°C. All the* in vivo* procedures were in accordance with the Italian legislative decree (D.L.) number 116 of January 27, 1992, and associates guidelines in the European Communities Council Directive of November 24, 1986 (8676097ECC). All efforts were made to minimize animal suffering and to reduce their number.

Rats were anaesthetized with urethane (10% wt/v; 10 mL/kg ip.) and placed on a surgical table. An arterial thrombus was induced by FeCl_3_ application onto the surface of the right carotid artery, as described by Kurz et al. [24]. The effect of topical application of IL-17A was also evaluated. In brief, following surgery, a piece of filter paper (Whatman n°1, 3 × 5 mm) soaked in 10 *μ*L of a solution of FeCl_3_ (5%) or in recombinant mouse IL-17A (100 *μ*g/mL in HCl 4 mM, R&D System, Abingdon, UK) was applied onto the external surface of the right carotid artery for 30 minutes. Afterward the paper was removed and the vessel was left* in situ* for 60 minutes, to enable thrombus formation. In another set of experiments, an IL-17A (100 *μ*g/mL), or vehicle (HCl 4 mM), soaked filter paper was applied on the vessel for 30 minutes before applying FeCl_3_ (5%), as described above. At the end of 60-minute period, a piece of about 2 cm in length of the right carotid artery (and its contralateral) was excised and weighed. Thrombus size was evaluated by the difference in weight between the treated vessel and its contralateral.

### 2.2. Morphological Analysis

In another group of animals, the experiment was performed as described above and a segment (2 cm) of each treated vessel was excised, rinsed in saline to remove the blood excess, and then fixed in formalin (4% v/v; Carlo Erba, Italy) for 24 hours. Samples were processed and embedded in paraffin. Sections (10 *μ*m thick) were then stained with haematoxylin and eosin (Carlo Erba, Italy) in order to be morphologically analyzed. In all cases, a minimum of 5 sections per animal were analysed by using a standard light microscope (×5 and ×10 objective). Images were taken by a Leica DFC320 video camera (Leica, Milan, Italy) connected to a Leica DM RB microscope using the Leica Application Suite software V2.4.0.

### 2.3. Western Blot Analysis of CD39

In subsets of experiments, following local treatment with IL-17A or with its vehicle (HCl 4 mM) for 30 minutes, as described above, CD39 expression was evaluated on carotid section homogenates by Western blot analysis. For this purpose, following local treatment, carotids were immediately frozen in liquid nitrogen before being stored at −80°C. On the day of analysis, tissues were homogenized using liquid nitrogen in the following lysis buffer: Tris-HCl pH 7.5, 50 mM; NaCl, 150 mM; sodium orthovanadate, 1 mM; *β* glycerophosphate, 20 mM; EDTA, 2 mM; PMSF 1 mM; leupeptin, 5 *μ*g/mL; aprotinin, 5 *μ*g/mL; pepstatin, 5 *μ*g/mL. Protein concentration was determined by the Bio-Rad protein assay kit (Bio-Rad, Italy). Protein samples (35 *μ*g) were subjected to electrophoresis on an SDS 8% polyacrylamide gel and transferred onto a nitrocellulose transfer membrane (Protran, Schleicher & Schuell, Germany). The membranes were saturated by incubation with nonfat dry milk (5% wt/v) in PBS supplemented with 0.1% (v/v) Tween 20 (PBS-T) for 1 h at room temperature and then incubated with a goat monoclonal anti-CD39 antibody (A-16, Santa Cruz, CA) (dilution 1 : 200), overnight at 4°C. Successively, membranes were washed and then incubated with the secondary antibody conjugated with horseradish peroxidase, anti-goat IgG-HRP (dilution 1 : 2000, Dako Denmark), for 2 h at room temperature. Protein bands were detected using the enhanced chemiluminescence (ECL) detection kit and Image Quant 400 GE Healthcare software (GE Healthcare, Italy). Protein bands were quantified using GS 800 imaging densitometer software (Biorad, Italy).

### 2.4. Enzyme Assay

On carotid section homogenates, ADP hydrolyzing activity was evaluated using a modification of the method described by Saucedo et al. [25]. The reaction medium used to assay ADPase activity contained 120 mM NaCl, 5.0 mM KCl, 60 mM glucose, 5 mM CaCl_2_, and 50 mM Tris-HCl buffer, pH 7.5 in a final volume of 200 *μ*L. A sample volume of 20 *μ*L (10 *μ*g proteins) was added to the reaction medium and preincubated for 10 minutes at 37°C. The enzyme reaction was then started by the addition of ADP at a final concentration of 2 mM and incubated for 40 minutes at 37°C. Controls were performed by adding deionized H_2_O to each sample, instead of ADP. The reaction was stopped by the addition of 200 *μ*L of trichloroacetic acid (TCA) 10%. Samples were then centrifuged at 3000 rpm × 10 min. As a measure of ADPase activity, inorganic phosphate (Pi) released was quantified by a colorimetric assay Sensolyte assay kit (AnaSpec) and expressed as nmol Pi released* per* *μ*g of protein [26].

### 2.5. Statistical Analysis

Data were expressed as mean ± S.E. and analysed by one way analysis of variance (ANOVA), followed by Bonferroni's test for multiple comparisons, or by unpaired two-tailed Student's* t*-test when appropriate. In some cases, one-sample* t*-test was used to evaluate significance against the hypothetical zero value. A value of *P* < 0.05 was taken as significant.

## 3. Results and Discussion

**Figure 1:**
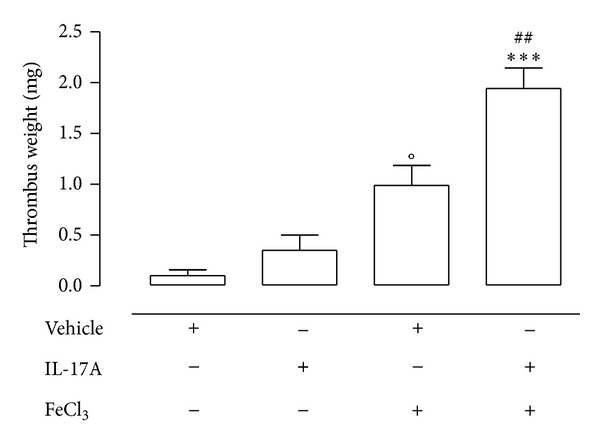
Effect of IL-17A on thrombus mass induced by FeCl_3_ application on rat carotid artery. A filter paper soaked in FeCl_3 _(5%) or in recombinant mouse IL-17A (100 *μ*g/mL) was applied onto the external surface of the right carotid artery, for 30 minutes; afterward the paper was removed and the vessel was left* in situ* for 60 minutes, to enable thrombus formation. In another set of experiments, an IL-17A (100 *μ*g/mL), or vehicle (HCl, 4 mM), soaked filter paper was applied onto the vessel for 30 minutes before applying FeCl_3_ (5%). At the end of 60-minute period, a piece of about 2 cm in length of the right carotid artery and of its contralateral was excised and weighed. Thrombus size was evaluated by the difference in weight between the treated vessel and its contralateral. All controls were performed by applying only vehicle (H_2_O or HCl 4 mM). ^##^ *P* < 0.01 versus FeCl_3_, ***P < 0.001 versus IL-17A, and °*P* < 0.05 versus vehicle (one way ANOVA followed by Bonferroni's test; *n* = 8–10).

The increased platelet reactivity, endothelial dysfunction, and atherosclerotic plaques instability are common features of subjects affected by systemic immunological inflammatory diseases and might represent the cause of the increased cardiovascular risk observed in these patients [1, 5, 23]. However, which factors are primarily responsible for predisposing to haemostasis disturbances and to cardiovascular events in chronic autoimmune diseases is still unknown. Recently, IL-17A, which is critically involved in the pathogenesis of autoimmune diseases, has been claimed as a possible candidate to participate in endothelial dysfunction and increased cardiovascular risk [19, 20]. On these bases, our work was aimed at evaluating* in vivo* the effect of IL-17A in a model of ferric chloride-induced arterial thrombosis. We found that application of FeCl_3_ (5%) caused an intravascular thrombus of 1.10 ± 0.23 mg (*n* = 9; *P* < 0.01 one-sample* t*-test) whose mass increased significantly (1.91 ± 0.23 mg; *n* = 8. *P* < 0.01) when carotid was pretreated with IL-17A (100 *μ*g/mL), 30 minutes before FeCl_3_ application. Application of IL-17A alone on the external surface of rat carotid artery generated* per se* a small intravascular thrombus (0.42 ± 0.17 mg; *n* = 10; *P* < 0.05) whereas nonsignificant effects were observed after vehicle exposure (0.1 ± 0.05 mg; *n* = 7) (Figure 1).

**Figure 2:**
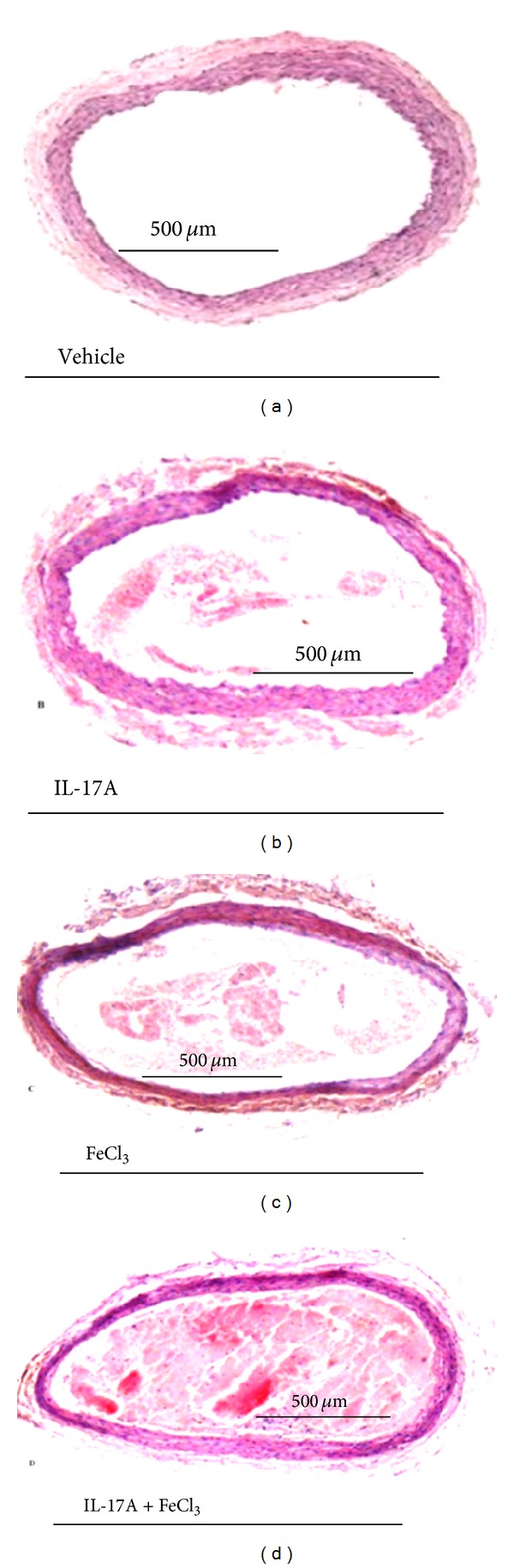
Photomicrographs showing hematoxylin and eosin histologic cross sections of carotid arteries following local treatment as described in the method section (magnification ×50). (a) Vehicle (HCl 4 mM); (b) IL-17A (100 *μ*g/mL); (c) vehicle (HCl 4 mM)* plus* FeCl_3 _(5%); and (d) IL-17A and FeCl_3_.

Morphological analysis of the vessel section evidenced that the luminal surface of carotid sections from control was covered by a continuous endothelium. Moreover the vascular lumen was not characterized by aggregates (Figure 2(a)). Interestingly, sections obtained from IL-17A* plus* FeCl_3_ showed an occluding thrombus compared with IL-17A (Figure 2(b)) or FeCl_3_ (Figure 2(c)) treated carotids. Furthermore, the endothelium appeared damaged and vessel wall thickness extremely reduced (Figure 2(d)).

It is known that arterial thrombosis is primarily caused by platelet adhesion and activation to the damaged vessel [21]. Following activation, endothelium loses its antithrombotic properties and platelets may become activated [2]. The model of FeCl_3_-induced thrombosis has been shown to involve platelets and several components of haemostasis [27], including TF [28].

**Figure 3:**
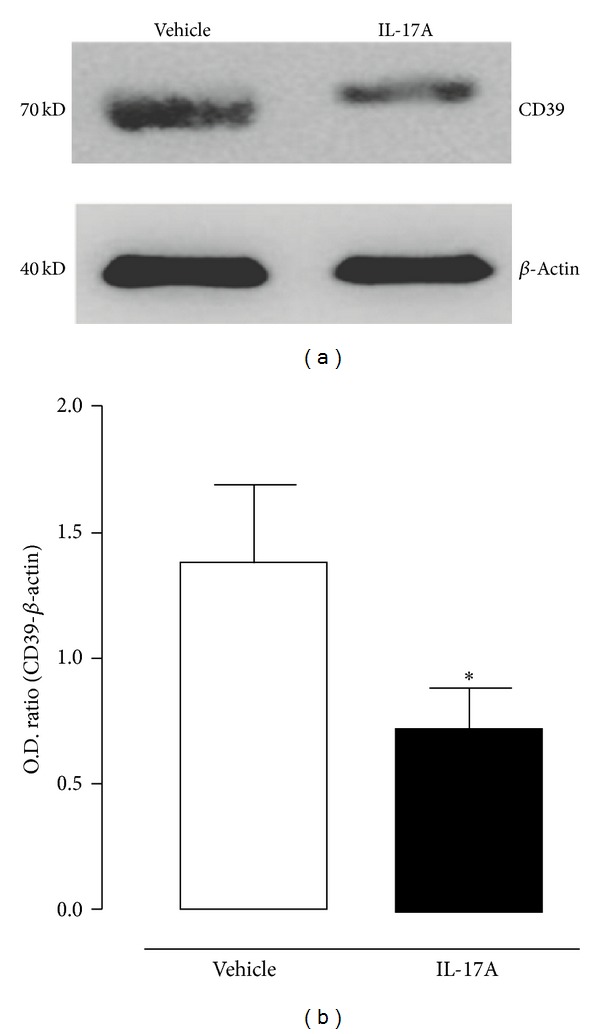
(a) Representative results of Western blot analyses of CD39 expression on rat carotids treated for 30 minutes with vehicle or IL-17A. (b) Densitometric analysis of Western blots; O.D. (optical density) normalized against *β*-actin. *P < 0.05 versus vehicle (Student's* t*-test; *n* = 3).

**Figure 4:**
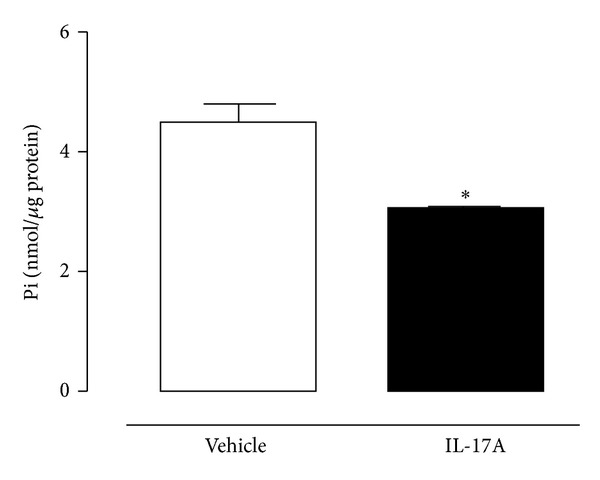
Quantification of inorganic phosphates (Pi) produced by rat carotid homogenates treated with vehicle or IL-17A. Pi produced was evaluated as measure of ADP hydrolyzing activity (for details, see method section). *P < 0.05 versus vehicle (Student's* t*-test; *n* = 3).

CD39/ATP diphosphohydrolase (ATPDase) is largely expressed on vascular endothelial cells and by converting ATP and ADP to monophosphate form (AMP) it represents a key modulator of vascular haemostasis and thrombogenesis. The loss of CD39 on activated endothelial cells causes platelet sequestration and TF upregulation, key events for thrombogenesis [29]. Nonetheless, CD39 also may offer protection against myocardial ischemia-reperfusion injury since it promotes ADP degradation and thus favours, in tandem with CD73 (ecto-5′-nucleotidase), adenosine accumulation that represents a cardiovascular protective molecule [30]. It was shown that mice lacking CD39 had vascular features of a prethrombotic state, characterized by fibrin deposition in several vascular beds [31]. Recently, it has been demonstrated that IL-17A reduces CD39 mRNA expression in human endothelial cells* in vitro* and, at the same time, it increases TF expression [20]. On these bases, and considering that CD39 plays a crucial role in maintaining the endothelium antithrombotic properties, we analysed its expression and its ADP hydrolyzing activity on rat carotid artery following the* in vivo* exposure to IL-17A. Results obtained show that following the application of IL-17A on carotid surface the expression of CD39 was significantly reduced compared with the application of the only vehicle (HCl, 4 mM) (Figure 3). Concomitantly, ADP hydrolyzing activity was also reduced as demonstrated by a reduced inorganic phosphate production, following incubation with ADP, from IL17A treated carotids compared to vehicle (Figure 4). Thus, through the downregulation of CD39, IL-17A might contribute to priming the vessel for the effect of a minimal FeCl_3_ concentration.

Our hypothesis would be consistent with the observation that IL-17A does not cause* per se* an intra-arterial occlusive thrombus, but it would induce those endothelial features peculiar to a prothrombotic state. Indeed, the lack of ADP hydrolysis due to CD39 downregulation on endothelial cells would not be a stimulus for thrombogenesis but, most likely, for the increase of platelet aggregates at the site of vascular injury and this would facilitate the effect of a thrombotic agent.

## 4. Conclusion

Our results give for the first time an* in vivo* evidence for a prothrombotic effect of IL-17A that is likely related to a downregulation of CD39 expression and activity in the vascular system. Accordingly with our previous results that demonstrated* in vitro* the effects of this cytokine on human and murine platelets [22], in this study we suggest that this cytokine might be an important molecule at the interface between haemostasis and inflammation. Intriguingly, the mechanistic bases of prothrombotic effect of IL-17A need further investigation.
